# Modelling of *BCS1L*-related human mitochondrial disease in *Drosophila melanogaster*

**DOI:** 10.1007/s00109-021-02110-1

**Published:** 2021-07-17

**Authors:** Michele Brischigliaro, Elena Frigo, Samantha Corrà, Cristiano De Pittà, Ildikò Szabò, Massimo Zeviani, Rodolfo Costa

**Affiliations:** 1 Department of Biology, University of Padova, Padova, Italy; 2 Department of Neurosciences, University of Padova, Padova, Italy; 3 Italian National Research Council (CNR) Institute of Neuroscience, Padova, Italy

**Keywords:** BCS1L, Mitochondrial disease, Mitochondrial respiratory chain, Drosophila melanogaster, Respiratory chain complex III

## Abstract

Mutations in *BCS1L* are the most frequent cause of human mitochondrial disease linked to complex III deficiency. Different forms of *BCS1L*-related diseases and more than 20 pathogenic alleles have been reported to date. Clinical symptoms are highly heterogenous, and multisystem involvement is often present, with liver and brain being the most frequently affected organs. *BCS1L* encodes a mitochondrial AAA + -family member with essential roles in the latest steps in the biogenesis of mitochondrial respiratory chain complex III. Since Bcs1 has been investigated mostly in yeast and mammals, its function in invertebrates remains largely unknown. Here, we describe the phenotypical, biochemical and metabolic consequences of *Bcs1* genetic manipulation in *Drosophila melanogaster.* Our data demonstrate the fundamental role of Bcs1 in complex III biogenesis in invertebrates and provide novel, reliable models for *BCS1L-*related human mitochondrial diseases. These models recapitulate several features of the human disorders, collectively pointing to a crucial role of Bcs1 and, in turn, of complex III, in development, organismal fitness and physiology of several tissues.

**Supplementary Information:**

The online version contains supplementary material available at 10.1007/s00109-021-02110-1.

## Introduction

Ubiquinol-cytochrome *c* oxidoreductase (also known as cytochrome *bc*_*1*_ or complex III) is the central electron transfer complex of the mitochondrial respiratory chain (MRC). It catalyses the oxidation of reduced coenzyme Q (CoQ) coupled with reduction of cytochrome *c*. The fully functional complex III is a dimer of the holoenzyme (complex III_2_), composed of three catalytic core subunits. One of them is mtDNA-encoded (MT-CYB) whereas two are nuclear (n)DNA-encoded (CYC1 and UQCRFS1) [2]. Eight additional subunits (UQCRC1, UQCRC2, UQCRH, UQCRB, UQCRQ, Subunit 9, UQCR10 and UQCR11) are not directly involved in catalysis but are however needed for the correct functioning of the enzyme. MT-CYB contains the two CoQ binding sites, Q_o_ and Q_i_, and two b-type heme groups with different redox potentials (low redox potential *b*_*L*_ and high redox potential *b*_*H*_). CYC1 harbours a c-type heme centre (*c*_*1*_) and UQCRFS1 (also known as Rieske Fe-S protein) contains a 2Fe-2S cluster. Complex III couples its redox reactions to proton pumping through the Q cycle [3].

The biogenesis of complex III has been extensively studied in yeast, and the underlying molecular mechanisms are emerging from different studies (reviewed by [5]). Moreover, it is now well known that the whole process requires a set of proteins, including enzymes and chaperones, which are not part of the complex but play a role in its biogenesis and assembly [5]. Among the complex III-associated assembly factors, BCS1L is the most studied. BCS1L is a member of the AAA + family (ATPases associated with diverse cellular activities), as it displays a typical AAA + domain. During complex III biogenesis, BCS1L acts at the later stages, carrying out the incorporation of the catalytic UQCRFS1 and the smallest supernumerary subunit UQCR11 into the complex [6–8]. Experimental evidence indicates that heptameric structures of BCS1L act on UQCRFS1 via protein translocation, promoting its insertion into the inner mitochondrial membrane (IMM) after being imported into the mitochondrial matrix [9–11].

Mutations in *BCS1L* are the most frequent forms of complex III deficiency in humans (OMIM no. 124000), [12]. The first pathogenic variants of *BCS1L* were described in 2001 [13]. Over the following two decades, several other mutant *BCS1L* alleles linked to complex III deficiency have been reported and associated with a set of mitochondrial disorders of varying severity, ranging from early onset, lethal diseases, to mild conditions with chronic clinical courses. For example, BCS1L founder variants restricted to Scandinavian areas cause GRACILE syndrome (growth retardation, aminoaciduria, cholestasis, iron overload, lactic acidosis and early death) [14, 15, 47], whereas others are associated with metabolic acidosis and liver failure [13, 16–20], sensorineural Björnstad syndrome [21, 22] or sensorineural hearing loss with developmental delay, hypotonia and encephalopathy [23].

To date, the only available animal model for a *BCS1L*-related disorder is a knock-in mouse carrying the GRACILE syndrome founder missense mutation c.232A > G (p.Ser78Gly). The homozygous mutant mouse displays reduced growth, lactic acidosis and liver impairment, progressing from steatosis to fibrosis, leading to death at approximately 6 weeks of age [24]. However, for yet unknown and controversial reasons, young animals have been reported to have normal Rieske Fe-S incorporation and complex III activity [24]. Interestingly, a recent report [25] has shown that the original GRACILE mouse resulted from the additional presence of a mtDNA mutation combined with the BCS1L c.232A > G (p.Ser78Gly) mutation, so that when the GRACILE and the mtDNA mutations are separated, the mouse phenotype is milder and lacks the features that are believed to cause infantile death in humans. Recently, the *bcs1l* ortholog in zebrafish has been implicated in warranting physiological mitochondrial respiration and contributing to normal morphogenesis [26]. Finally, a yeast strain carrying a single point mutation (F342C) in the Bcs1-specific domain has also been reported [27].

Here, we describe the generation and characterization of the second metazoan model of *BCS1L-*related mitochondrial disease. Genetic tools were utilised to explore the phenotypical, molecular and biochemical consequences of the loss of the BCS1L homolog in the invertebrate *Drosophila melanogaster*, defining the homeostatic role of the protein—most likely as a cytochrome *bc*_*1*_ assembly factor—in development and physiology of several tissues.

## Results

### *CG4908* is the *D. melanogaster* ortholog of human *BCS1L*

**Fig. 1:**
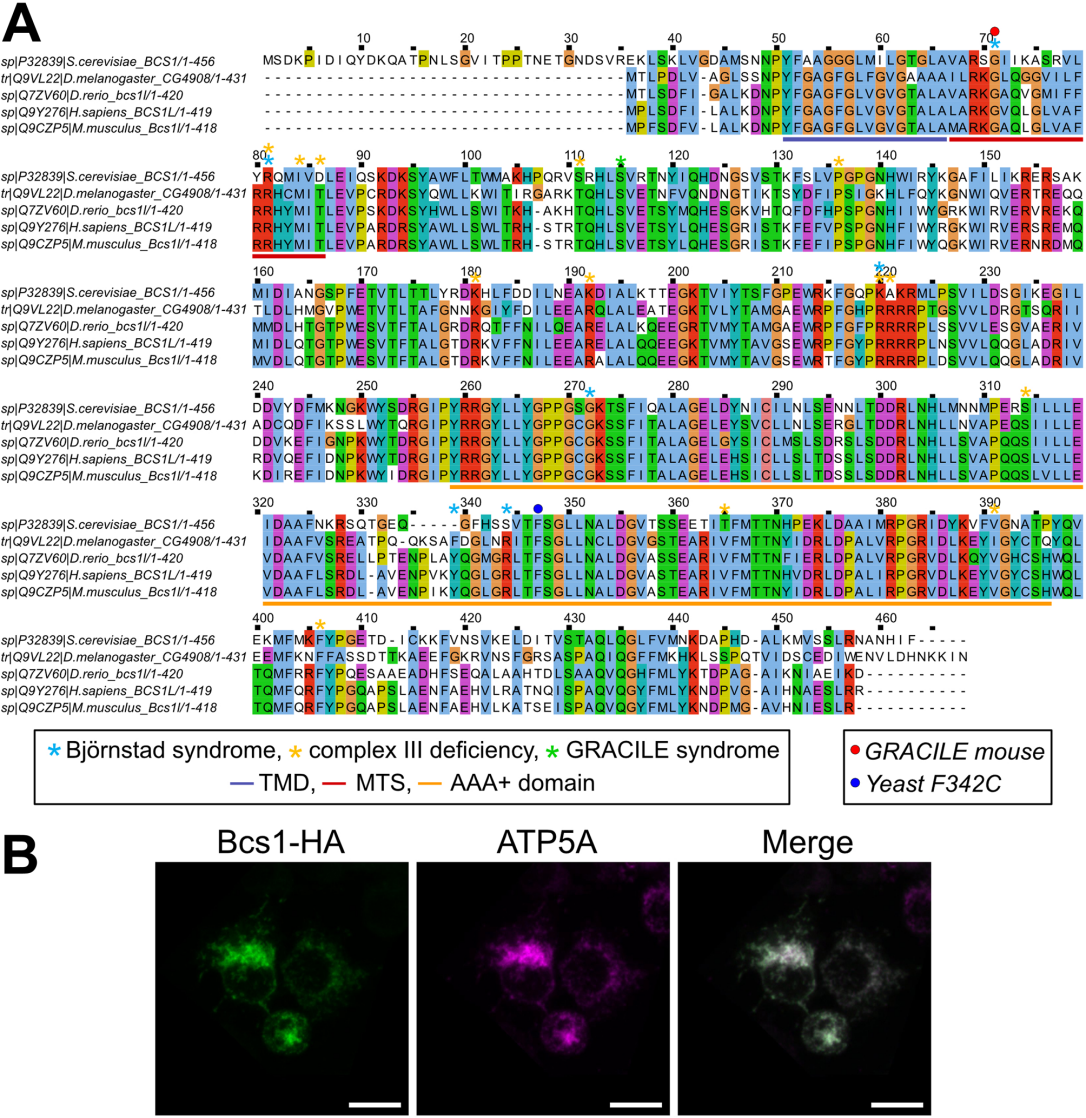
*CG4908* is the *D. melanogaster* ortholog of human *BCS1L*. **A** Global alignment of fruitfly’s Bcs1 and human BCS1L proteins. Conserved domains are marked by coloured lines below the alignment, TMD by the blue line, internal MTS by the red line and AAA + ATPase domain by the yellow line. Mutated sites found in patients are indicated with stars above the sequence; Björnstad syndrome sites are marked by blue stars, complex III deficiency sites by yellow stars and the GRACILE syndrome site by a green star. The red dot shows the GRACILE mutation (S78G) modelled in mouse and the blue dot shows the yeast F342C mutation. **B** Subcellular localization of *Drosophila* Bcs1. Confocal micrographs showing *Drosophila* cells expressing Bcs1-HA (green immunofluorescence, Alexa Fluor 488), and immunolabelled with anti-ATP5A antibody to mark mitochondrial structures (purple immunofluorescence, Alexa Fluor 647). Sum intensity projections (z-stacks) are shown. Scale bars: 10 μm

The integrative orthology prediction tool DIOPT [28] assigns the protein coding gene *CG4908* (hereafter named *Bcs1* and Bcs1 protein) as the high score putative *Drosophila melanogaster* ortholog of *Homo sapiens BCS1L*. Two transcripts of *Bcs1*, sharing the CDS, encode the same 431 amino-acid protein (Bcs1). Global alignment of *Drosophila melanogaster* Bcs1 and *Homo sapiens* BCS1L revealed a high degree of conservation of the protein sequence (Fig. 1A). Indeed, 60% of the protein sequence was found to be identical between human BCS1L and *D. melanogaster* Bcs1, and three domains of the protein known to be crucial for its localization and function—the N-terminal transmembrane domain (TMD), the internal mitochondrial targeting sequence (MTS) and the C-terminal AAA + ATPase domain—are highly conserved (Fig. 1A). Importantly, almost all the sites known to be mutated in patients with *BCS1L-*related mitochondrial disease [13–23, 47] are conserved in both species, and most of them are located into the three main domains (Fig. 1A). Both yeast and mammalian Bcs1 proteins were shown to be mitochondrial proteins, specifically located into the IMM [29, 30]. Since the subcellular localization of *Drosophila* Bcs1 has never been experimentally tested and validated, we cloned *CG4908* cDNA into the pAc5-STABLE2-neo expression vector with a HA-tag at the C-terminus (Bcs1-HA). Confocal imaging of immunostained S2R + cells using an antibody against HA-tag plus an antibody against a mitochondrial complex V subunit (ATP5A) lets us experimentally validate that *Drosophila melanogaster BCS1L-*homolog is targeted to mitochondria (Fig. 1B).

#### Partial loss of *Bcs1* in the whole organism has detrimental effects on flies’ development due to severe complex III deficiency

**Fig. 2:**
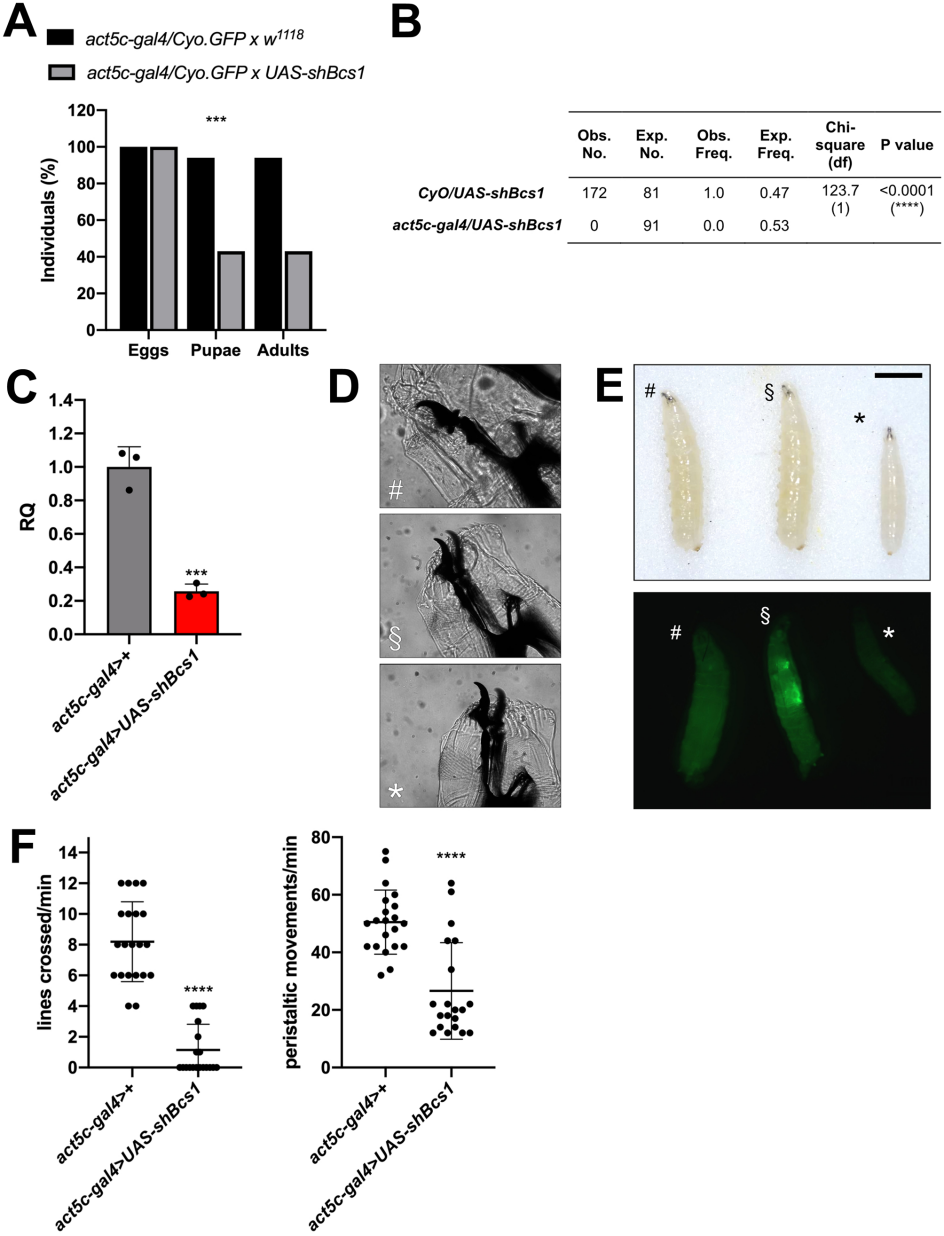
Ubiquitous KD of *Bcs1* in *D. melanogaster*. **A** Relative percentage of egg to adult viability of ubiquitous *Bcs1* KD cross (*act5c-gal4* > *CyO.GFP x UAS-shBcs1*) and control cross (*act5c-gal4* > *CyO.GFP x w*^*1118*^), calculated at three developmental stages (eggs, pupae and adults), (n > 250, chi-square test 16.80 df(2), ***p < 0.001). **B** Mendelian frequencies of adults obtained by crossing heterozygous *act5c-gal4* > *CyO.GFP* flies with homozygous *UAS-shBcs1* flies (n = 172, chi-square test 123.7, df(1), ****p ≤ 0.0001). **C** *Bcs1* expression levels normalized by *Rp49* reference expression levels and measured by qPCR in whole larvae, expressed as relative quantity of template in the sample (RQ) in ubiquitous KD larvae (*act5c-gal4* > *UAS-shBcs1*) compared to control larvae (*act5c-gal4* > +). Data are plotted as mean ± S.D. (n = 3, Student’s t test ***p ≤ 0.001). **D** Morphological analysis of mouth hooks in whole-mount larval preparations. #*act5c-gal4* > + control larvae; §*UAS-shBcs1* > *CyO.GFP* control larvae; *act5c-gal4 > *UAS-shBcs1* KD larvae. **E** Morphological evaluation of *Bcs1* KD larvae. #*act5c-gal4* > + control larvae; §*UAS-shBcs1* > *CyO.GFP* control larvae; *act5c-gal4 > *UAS-shBcs1* KD larvae. Scale bar: 1 mm. **F** Locomotor activity analysis of *Bcs1* KD larvae. Right = peristaltic movements per minute in larvae; left = number of lines crossed per minute by larvae (on a 0.25 cm^2^ grid). Genotypes: *act5c-gal4* > + control larvae and *act5c-gal4* > *UAS-shBcs1* KD larvae (n = 3, Student’s t test ****p ≤ 0.0001)

We exploited the binary system *UAS/GAL4* [31] to drive the post-transcriptional silencing of *Bcs1* in *Drosophila*. Firstly, the *act5c-gal4* construct was used to drive ubiquitous *Bcs1* KD. Crossing of heterozygous *act5c-gal4/CyO.GFP* with *UAS-shBcs1* homozygous flies resulted in the loss of about 50% of the progeny between the larval and the pupal stage, and in altered mendelian ratios. All the individuals reaching the pupal and the adult stages were heterozygous *UAS-shBcs1/CyO.GFP* and therefore unsilenced (Fig. 2A, B). In fact, *act5c-gal4 >* *UAS-shBcs1* larvae were found in the progeny and screened by qPCR, documenting a loss of about 80% of the transcript in KD larvae compared to control larvae (Fig. 2C). This resulted in complete egg-to-adult lethality, with KD larvae failing to thrive and dying at the 3rd larval stage, as documented by the presence of fully developed mouth hooks (Fig. 2D). *Bcs1* KD larvae were also smaller than controls (Fig. 2E) and showed significant locomotor defects, including lower motility and dramatically decreased peristaltic movements compared to control larvae (Fig. 2F).

**Fig. 3:**
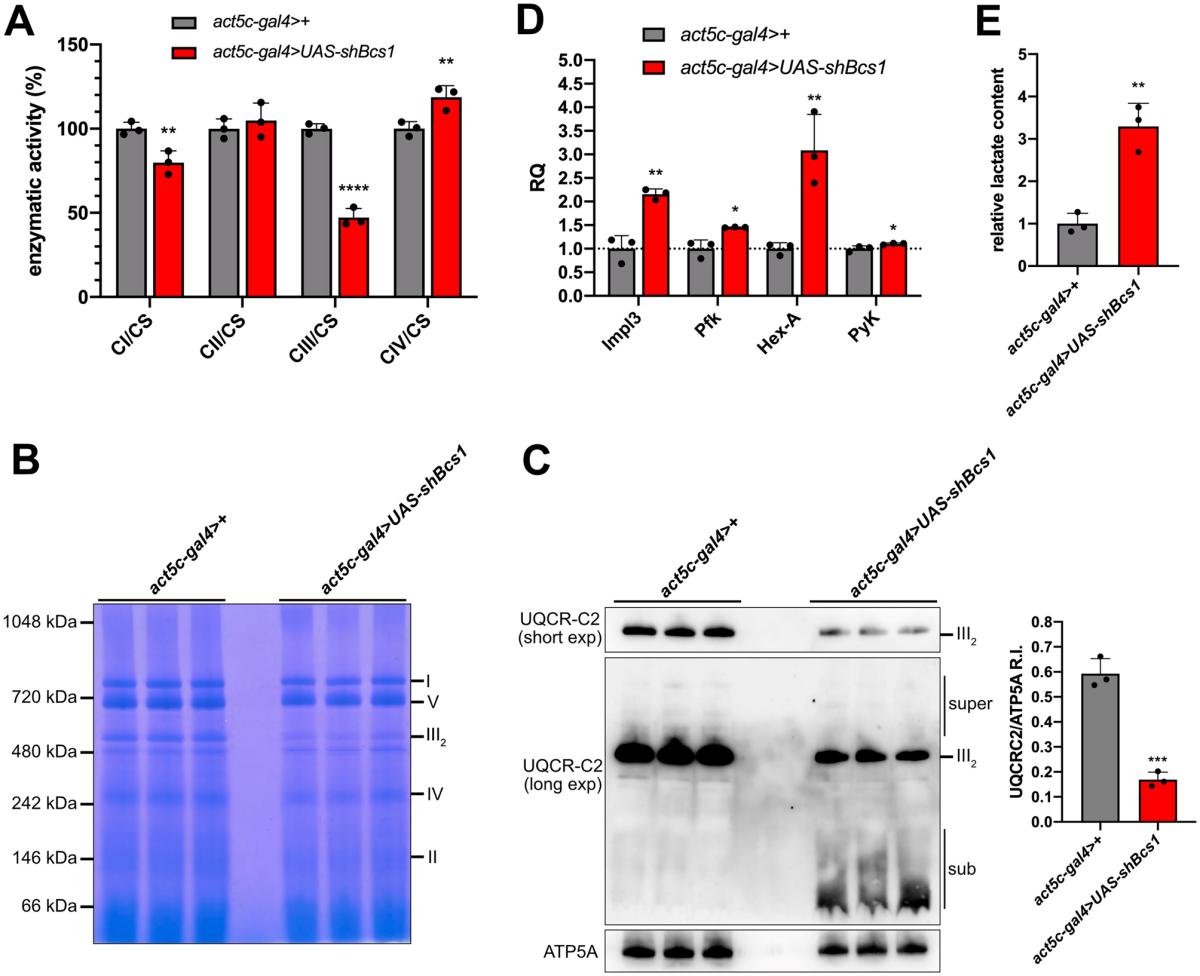
Biochemical characterization of *Bcs1* KD larvae. **A** Enzymatic activities of MRC complexes (I–IV) were measured in parental control (*act5c-gal4* > + , gray columns) and in ubiquitous KD larvae (*act5c-gal4* > *UAS-shBcs1*, red column). Activities of MRC complexes were normalized to the activity of citrate synthase (CS). For each genotype, three biological replicates of mitochondrial preparations were analysed. Data are plotted as mean ± S.D. (n = 3, Student’s t test, **p ≤ 0.01, ****p ≤ 0.0001). **B** Blue-native gel electrophoresis (BNGE) analysis of MRC complexes in DDM-solubilized isolated mitochondria from control (*act5c-gal4* > +) and *Bcs1* KD larvae (*act5c-gal4* > *UAS-shBcs1*). **C** BNGE, Western blot and immunodetection of DDM-solubilized mitochondria from *Bcs1* KD and control flies with antibodies against a CIII subunit (UQCR-C2) and a CV subunit (ATP5A) as loading control. Bands were quantified by measuring the integrated density of the signal. Data are plotted as mean ± S.D. (n = 3, Student’s t test ***p ≤ 0.001). **D** *Impl3* (*Ldh* homolog), *Pfk* (phosphofructokinase), *Hex-A* (hexokinase A) and *PyK* (pyruvate kinase) expression levels measured by qPCR in control (*act5c-gal4* > +) and *Bcs1* KD larvae (*act5c-gal4* > *UAS-shBcs1*), expressed as relative quantity of template in the sample (RQ) compared to control. Data plotted are mean ± S.D. (n = 3, Student’s t test, *p ≤ 0.05; **p ≤ 0.01). **E** Quantification of total body lactate in control (*act5c-gal4* > +) and *Bcs1* KD larvae (*act5c-gal4* > *UAS-shBcs1*), normalized to the protein content and expressed as relative quantity in the sample compared to control. Data are plotted as mean ± S.D. (n = 3, Student’s t test **p ≤ 0.01)

Biochemical analyses of MRC enzymatic activities revealed a severe reduction in CIII activity (47% of control activity) with a small but significant reduction in NADH/ubiquinone oxidoreductase (CI) activity (79% of control activity) (Fig. 3A). We also observed an unexpected, small but significant increase in cytochrome *c* oxidase (CIV) activity in *Bcs1* KD larvae (118% of control activity) (Fig. 3A). In addition, BNGE of DDM-solubilized mitochondrial complexes showed that the functional, dimeric form of CIII (CIII_2_) was predominantly and severely affected in *Bcs1* KD larvae (Fig. 3B). These results were further corroborated by immunovisualization of the BNGE of DDM-solubilized mitochondria from *Bcs1* KD and control larvae using an antibody against cytochrome *bc*_*1*_ core subunit 2 (UQCR-C2). As shown in Fig. 3C, Western blot analysis revealed severe reduction of the signal corresponding to CIII_2_ in *Bcs1* KD mitochondria. Furthermore, the accumulation of low-molecular–weight, UQCR-C2-containing sub-assembly species was detected in *Bcs1* KD individuals, as also previously observed in yeast and humans. In order to validate the specificity of the effects of *Bcs1* silencing on developmental arrest and CIII stability, we also analysed a different *Bcs1* UAS-RNAi line (*UAS-Bcs1-IR*) expressing a 344-bp inverted repeated sequence of *CG4908.* When the *UAS-Bcs1-IR* homozygous flies were crossed with heterozygous *act5c-gal4* > *CyO.GFP*, we observed a loss of about 50% of the progeny between the pupal and adult stages (figure S1A). Accordingly, we also observed altered mendelian ratios in the adults, as all of the progeny carried the *CyO* balancer chromosome (figure S1B), as in the cross between the *act5c-gal4* > *CyO.GFP* and the *UAS-shBcs1* lines. The size of GFP negative (*act5c-gal4* > *UAS-Bcs1-IR*) larvae from the progeny was comparable to that of both *act5c-gal4* > + and *UAS-Bcs1-IR* > *CyO.GFP*. Notably, when KD efficiency in *act5c-gal4* > *UAS-Bcs1-IR* larvae was assessed by qPCR, *Bcs1* expression levels were reduced by 57% compared to control larvae (S1C). Importantly, prior to further characterizing this second *Bcs1* RNAi line, we checked whether the off-target gene (named *Hoe2*) reported in the VDRC database entry for line *UAS-Bcs1-IR* was affected by the expression of the *Bcs1-IR* sequence. No alterations were observed in *Hoe2* expression (figure S1D). Next, by analysing the MRC of *Bcs1-IR* KD larvae by BNGE and immunoblot of BNGE as for the *act5c-gal4* > *UAS-shBcs1* KD line, *Bcs1-IR* KD larvae were found to have CIII_2_ deficiency (figure S1E), associated with accumulation of sub-assembly CIII_2_ species (figure S1F), similar to *shBcs1* KD larvae. The fact that two independent RNAi lines for *Bcs1* shared phenotypical and biochemical features suggests that these are indeed specifically linked to defects in *Bcs1* expression. Further, the two UAS-RNAi lines resulted in slightly different KD efficiencies (~ 80% by *UAS-shBcs1* construct and ~ 60% by *UAS-Bcs1-IR* construct) which, in turn, correlated with phenotypical traits (larval vs pupal lethality, respectively) and with the severity of CIII deficiency.

Lastly, we observed that *Bcs1* KD larvae have a strongly increased glycolytic metabolism in response to mitochondrial dysfunction linked to altered biogenesis and stability of CIII, as suggested by a twofold increase in *Impl3* (ortholog of mammalian lactate dehydrogenase, *Ldh*) expression levels and a threefold increase in total body lactate content (Fig. 3D, E). Accordingly, the expression levels of other three key enzymes of glycolysis (i.e., phosphofructokinase—*Pfk*, hexokinase A—*Hex-A* and pyruvate kinase—*PyK*) were significantly increased in *Bcs1* KD larvae (Fig. 3D). Taken together, these data demonstrate that physiological levels of *Bcs1* expression are essential for the developmental stages of *Drosophila*, and their loss is linked to altered biogenesis of MRC CIII_2_, mitochondrial dysfunction and metabolic switch from oxidative to glycolytic ATP production, causing lactate accumulation.

### Specific silencing of *Bcs1* in brain and dopaminergic neurons causes encephalopathic phenotypes and neuromotor dysfunction in flies

**Fig. 4:**
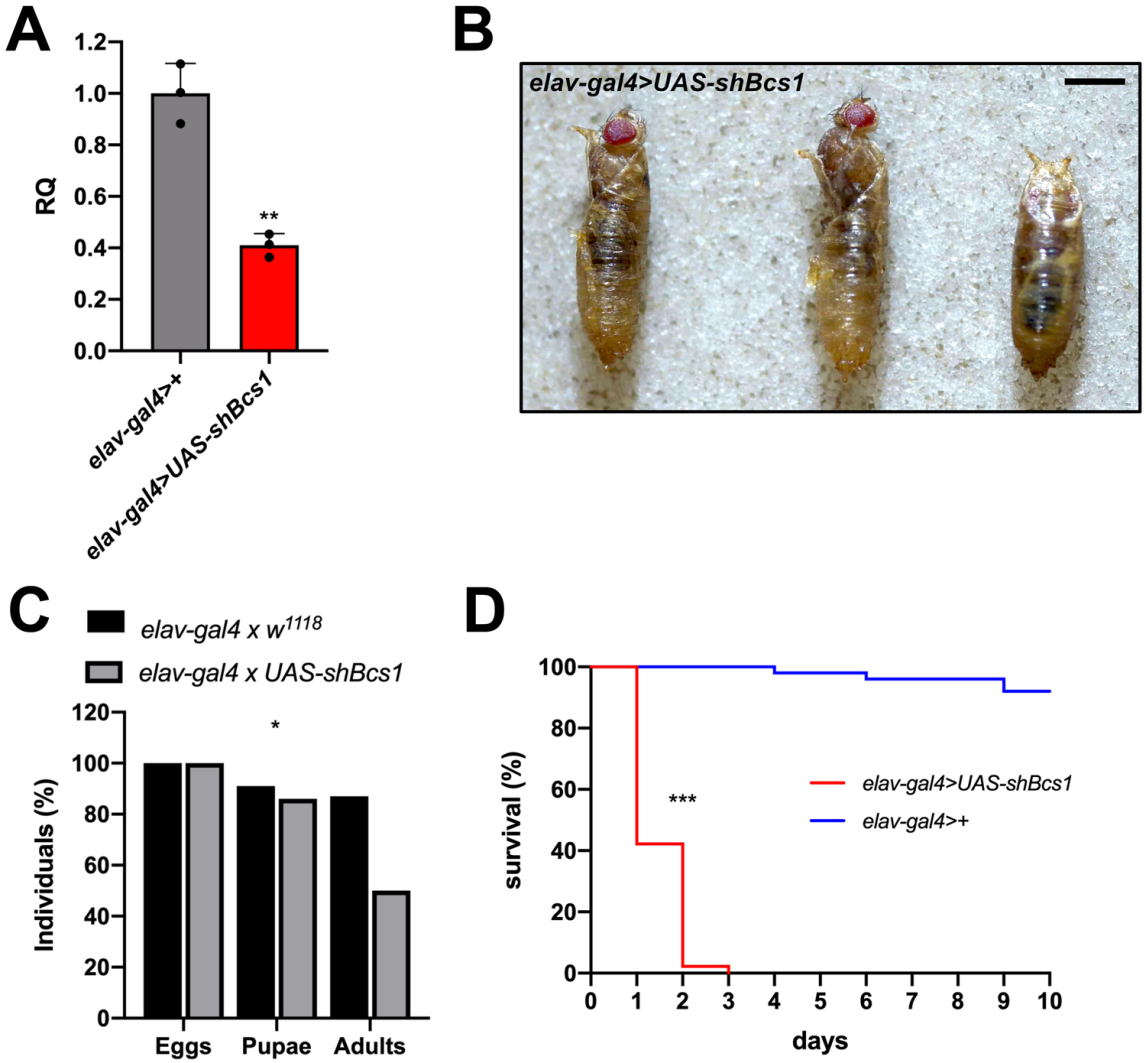
Pan-neuronal KD of *Bcs1* in *D. melanogaster*. **A** *Bcs1* expression levels normalized by *Rp49* reference expression levels and measured by qPCR in fly heads, expressed as relative quantity of template in the sample (RQ) in pan-neuronal KD larvae (*elav-gal4* > *UAS-shBcs1*) compared to control larvae (*elav-gal4* > +). Data are plotted as mean ± S.D. (n = 3, Student’s t test **p ≤ 0.01). **B** Morphological evaluation of pan-neuronal *Bcs1* KD individuals hatching from the pupae. Scale bar: 1 mm. **C** Relative percentage of egg to adult viability of pan-neuronal *Bcs1* KD cross (*elav-gal4 x UAS-shBcs1*) and control cross (*elav-gal4 x w*^*1118*^), calculated at three developmental stages (eggs, pupae and adults), (n > 250, Chi-square test 6.747 df(2), *p < 0.05). **D** Representative survival curves (Kaplan–Meier) of pan-neuronal control (*elav-gal4* > + , blue line) and KD (*elav-gal4* > *UAS-shBcs1*, red line) flies under standard culture conditions. Statistical significance was tested by the log-rank (Mantel-Cox) test (***p ≤ 0.001)

Given the strong phenotype observed in ubiquitous KD individuals, we sought to overcome developmental lethality through the tissue-specific silencing of *Bcs1*, by specifically knocking-down gene expression in fly homolog tissues that are mostly affected in patients, such as the brain. The CNS-specific knockdown mediated by crossing homozygous *UAS-shBcs1* flies with homozygous *elav-gal4* flies resulted in the reduction of 60% of *Bcs1* expression in the head (Fig. 4A). Knockdown flies were affected by partial pupal lethality, with nearly 50% of the expected adults missing. In fact, numerous fully developed individuals failed to emerge from the puparium, and some of them got stuck while hatching, eventually dying (Fig. 4B).

**Fig. 5:**
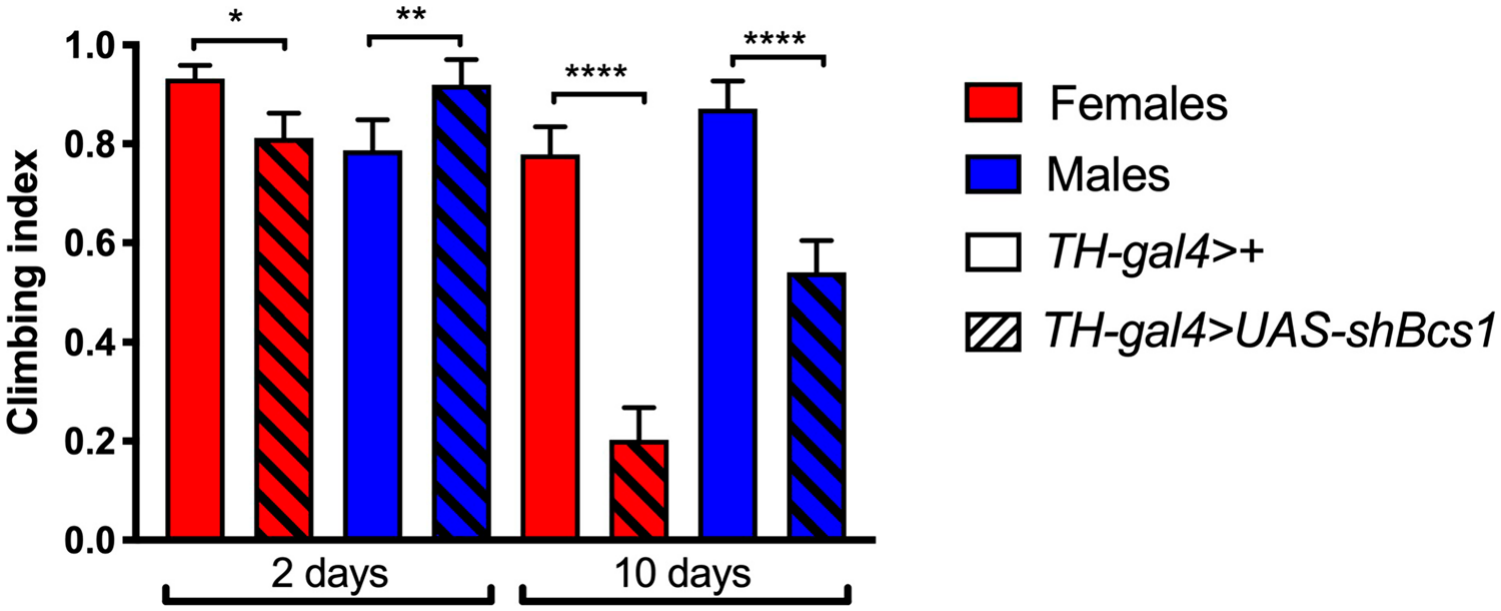
Neuromotor function in dopaminergic-specific *Bcs1* KD flies. Climbing performance of dopaminergic control (*TH-gal4* > +) and *Bcs1* KD (*TH-gal4* > *UAS-shBcs1*) female (red bars) and male (blue bars) flies at two different ages (2 and 10 days after hatching). Charts show mean and 95% CI, n = 60 animals. Statistical analysis was performed with one-way ANOVA with Dunn’s multiple comparisons test (*p ≤ 0.05, **p ≤ 0.01; ****p ≤ 0.0001)

Notably, all the hatched individuals (Fig. 4C) were unable to stand or walk and displayed paralysis, with seizures and dystonic movements (movie 1). Furthermore, escapers died within 3 days after hatching (Fig. 4D), most likely for inability to feed. Interestingly, the specific KD of *Bcs1* in the dopaminergic neurons (via *TH-gal4*) was sufficient to trigger a neurodegenerative phenotype in aging flies. Indeed, 10-day-old flies displayed neuromotor disability, i.e. strong reduction in climbing performance (26% and 62% of control scores in females and males, respectively), which was not observed in young individuals (Fig. 5). Our results indicate that females are more susceptible than males to *Bcs1* defects in the dopaminergic neurons. Collectively, these observations demonstrate that maintaining physiological levels of *Bcs1* expression and function in the CNS is essential to preserve neuromotor function and viability in *Drosophila* and shows that knocking-down *Bcs1* expression in the specific *TH-*positive neuronal population is sufficient to alter the behaviour of the flies, leading to age-specific neuromotor dysfunction.

### Specific silencing of *Bcs1* in muscle causes myopathic phenotypes flies

**Fig. 6:**
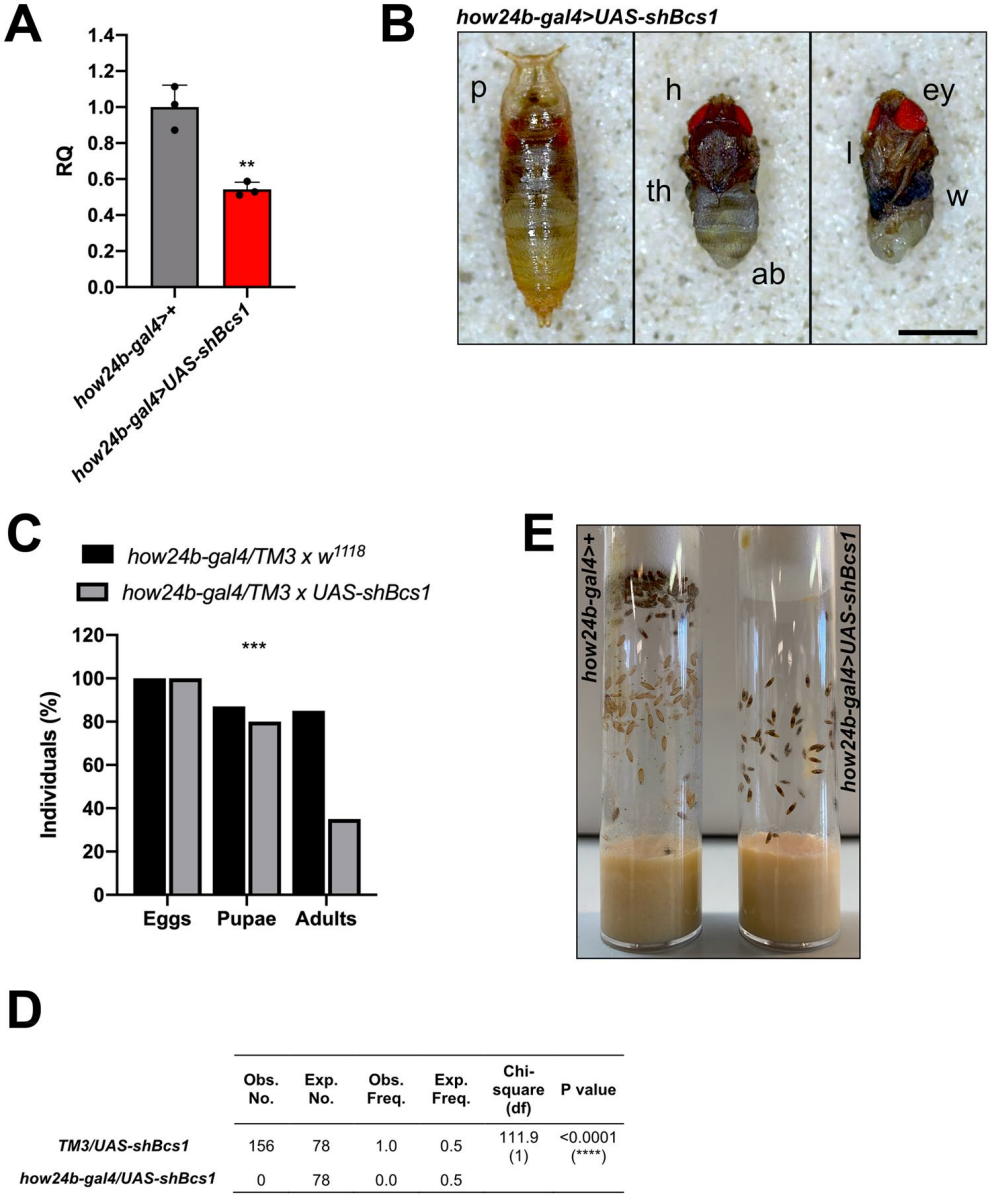
Muscle-specific KD of *Bcs1* in *D. melanogaster.* **A** *Bcs1* expression levels normalized by *Rp49* reference expression levels and measured by qPCR in larval body wall muscles, expressed as relative quantity of template in the sample (RQ) in muscle-specific KD larvae (*how24b-gal4* > *UAS-shBcs1*) compared to control larvae (*how24b-gal4* > +). Data are plotted as mean ± S.D. (n = 3, Student’s t test **p ≤ 0.01). **B** Morphological evaluation of muscle-specific *Bcs1* KD pupae. p = pupa, h = head, th = thorax, ab = abdomen, ey = eyes, l = legs, w = wings Scale bar: 1 mm. **C** Relative percentage of egg to adult viability of muscle-specific *Bcs1* KD cross (*how24b-gal4/TM3 x UAS-shBcs1*) and control cross (*how24b-gal4/TM3 x w*^*1118*^), calculated at three developmental stages (eggs, pupae and adults) (n > 250, Chi-square test 14.66 df(2), ***p < 0.001). **D** Mendelian frequencies of adults obtained by crossing homozygous *how24b-gal4* flies with homozygous *UAS-shBcs1* flies (n = 156, Chi-square test 111.9, df(1), **** p ≤ 0.0001).**E** Representative image of the progeny obtained by crossing homozygous *how24b-gal4* flies with homozygous *UAS-shBcs1* flies. Control flies (left vial) hatch from the pupa and reach the adult stage whereas muscle-specific *Bcs1* KD flies (right vial) fail to hatch and die at the latest pupal stage

The striking phenotypes resulting from CNS-specific KD of *Bcs1* prompted us to downregulate *Bcs1* expression in fly mesodermal derivatives, using the *how24b-gal4* driver. After crossing heterozygous *how24b-gal4* > *TM3* with homozygous *UAS-shBcs1*, we detected a 50% reduction by qPCR in *Bcs1* expression in the larval body wall muscles (Fig. 6A). Although almost all KD larvae grew and reached the pupal stage, fully developed individuals (Fig. 6B) failed to hatch (Fig. 6C), even over several days. Indeed, all the adult individuals in the progeny were *UAS-shBcs1* > *TM3* (Fig. 6D). Remarkably, crossing of homozygous *how24b-gal4* with homozygous *UAS-shBcs1* flies confirmed the observed lethality, with the total absence of adults in the progeny (Fig. 6E). Thus, muscle-specific KD of *Bcs1* in *Drosophila* leads to lethality at the latest pupal stages, likely due to inability of the flies to perform muscle contractions and coordinate movements needed to hatch.

### *Bcs1* silencing in the fat body has minor effects on organismal fitness in flies

**Fig. 7:**
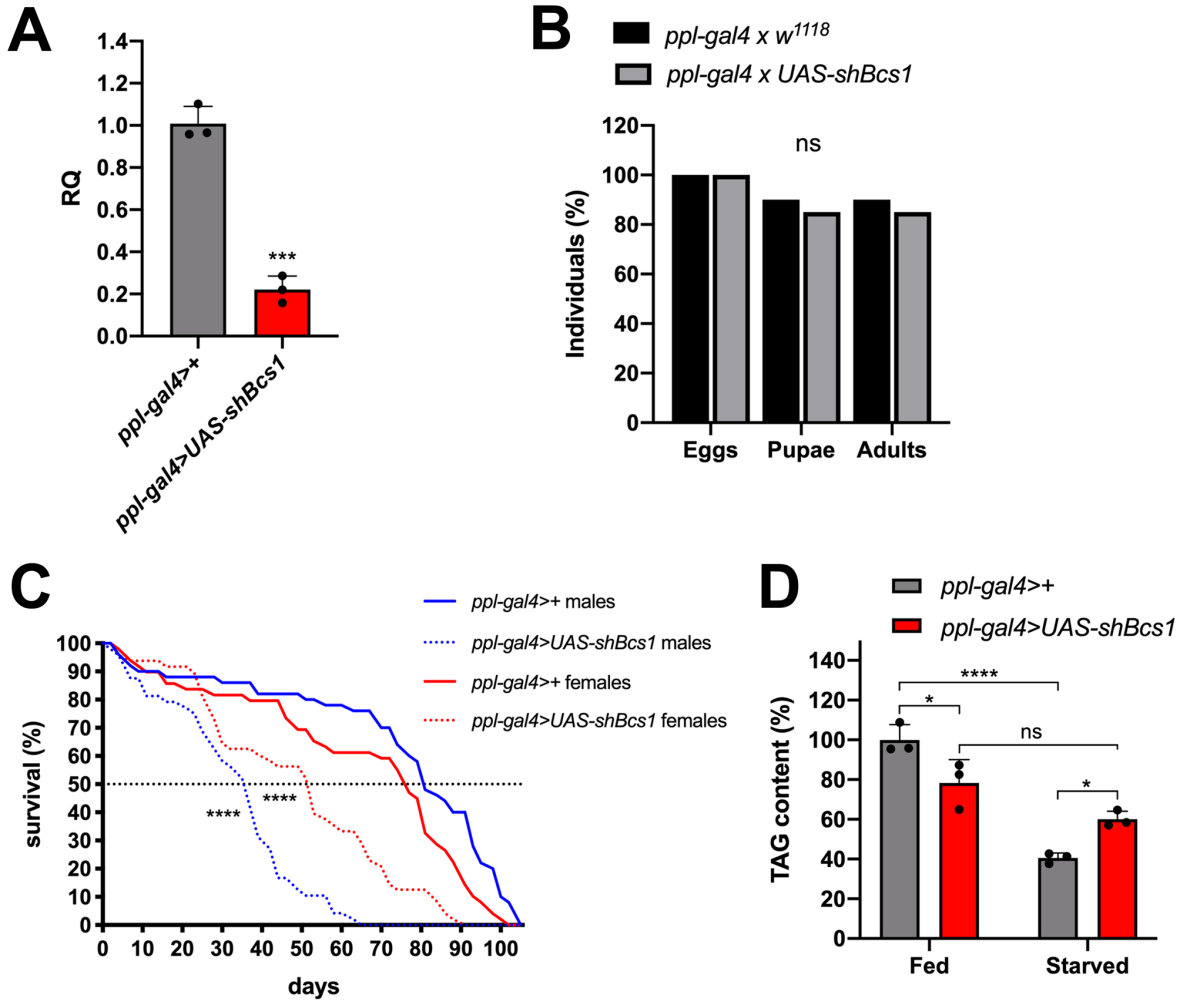
Fat body–specific KD of *Bcs1* in *D. melanogaster*. **A** *Bcs1* expression levels normalized by *Rp49* reference expression levels and measured by qPCR in adult abdominal fat body, expressed as relative quantity of template in the sample (RQ) in fat body–specific KD adults (*ppl-gal4* > *UAS-shBcs1*) compared to control adults (*ppl-gal4* > +). Data are plotted as mean ± S.D. (n = 3, Student’s t test ***p ≤ 0.001). **B** Relative percentage of egg to adult viability of fat body–specific *Bcs1* KD cross (*ppl-gal4 x UAS-shBcs1*) and control cross (*ppl-gal4 x w*^*1118*^), calculated at three developmental stages (eggs, pupae and adults), (n > 250, chi-square test 0.1039 df(2), ns = not significant. **C** Representative survival curves (Kaplan–Meier) of fat body control (*ppl-gal4* > + , solid lines) and KD (*ppl-gal4* > *UAS-shBcs1*, dotted lines) flies under standard culture conditions. Females and males are represented by red and blue lines, respectively. Statistical significance was tested by the log-rank (Mantel-Cox) test (****p ≤ 0.0001). **D** Quantification of TAG in control (*ppl-gal4* > +) and *Bcs1* KD abdominal fat body (*ppl-gal4* > *UAS-shBcs1*), normalized to the protein content and expressed as relative quantity in the sample compared to control. Data are plotted as mean ± S.D. (n = 3, two-way ANOVA with Tukey’s multiple comparisons ns = not significant, *p ≤ 0.05, ****p ≤ 0.0001)

Since the most severe cases of *BCS1L-*related mitochondrial diseases affect liver function, mostly leading to fatal metabolic syndromes, we knocked-down *Bcs1* expression in the fat body, the fly functional analogue of liver and white adipose tissue, by crossing homozygous *ppl-gal4* flies with homozygous *UAS-shBcs1* flies. Although the expression of *Bcs1* was drastically reduced (20% of the control) in the abdominal fat body of the progeny (Fig. 7A), the specific KD of the gene by *ppl-gal4* did not lead to major effects during development, since similar to controls, a high percentage of laid eggs (85% vs 90% in control cross) reached the adult stage (Fig. 7B). However, we observed reduced lifespan in *Bcs1* KD flies, with median survival rates between 44 and 67% of controls in male and female flies, respectively (Fig. 7C). We also detected impaired β-oxidation by quantifying the lipid content (triacylglycerides, TAGs) in the fat body of the adults (Fig. 7D). Indeed, in spite of having slightly but significantly lower TAG content in the fat body under normal feeding conditions compared to controls, *Bcs1* KD adults retained higher content of TAG after 24 h of starvation, when mobilization of fatty acids from TAG is needed to fuel β-oxidation, preserving oxidative metabolism (Fig. 7D). Taken together, these results suggest that in the fat body of invertebrates, *Bcs1* has a dispensable role during development but is essential to preserve mitochondrial β-oxidation and viability in the adult stage.

## Discussion

Among human mitochondrial diseases, complex III deficiency is one of the rarest. Over the past 20 years, the *BCS1L* gene has emerged as the main cause of complex III deficiency, leading to conditions such as GRACILE syndrome, Björnstad syndrome and severe metabolic syndromes [12]. Yeast has provided important information on the role of Bcs1 in the incorporation of the catalytic Rieske Fe-S subunit into complex III [6, 8] and work on human disease in vitro models has confirmed this function [7]. Other studies have elucidated the molecular mechanism of UQCRFS1 loading into complex III by BCS1L, proposing and refining the model by which a homo-heptamer of BCS1L mediates the process by ATP-driven pushing of UQCRFS1 across the membrane into the pre-complex III [9–11]. While providing useful information on the molecular mechanisms underlying genetic defects in eukaryotes, yeast has obvious limitations as a model of human clinical conditions. In 2010, the first mouse model harbouring the GRACILE syndrome founder mutation in *BCS1L* was described [24]. As already mentioned, these animals recapitulate some of the clinical features of patients, but they are also very different from patients, especially for their baffling biochemical and molecular features, which appear normal at young age [24, 32]. Young mice fail to display overt defects in complex III activity, and steady-state levels of UQCRFS1 are comparable to those of controls. By contrast, older, symptomatic animals start displaying the typical features of complex III deficiency that characterize the human condition [24].

Since *Drosophila melanogaster* harbours an uncharacterized homolog of BCS1L in its genome (*Bcs1*), we decided to expand the set of *BCS1L-*related mitochondrial disease models by generating models of *Bcs1* deficiency in flies. Firstly, we were able to show that the protein is highly conserved, especially in domains that are known to be essential for its function. This analysis also highlighted that most of the sites affected by missense mutations are conserved between humans and flies. Further, we demonstrated that Bcs1 protein is a *bona fide* mitochondrial protein in *Drosophila* cells. Regarding the effects of dysfunctional *Bcs1* in flies, we exploited the *UAS/GAL4* binary system in order to drive the silencing of the gene by expressing a specific shRNA in different tissues and systems. We showed that the loss of most but not all of the transcript in the whole organism causes developmental arrest between the third larval and the pupal stages and failure to reach the adult stage. This correlates with higher expression of key enzymes of the glycolytic pathway (*Ldh*, *Pfk*, *Hex-A*, *PyK)*, together with a dramatic increase in total body lactate in larvae lacking physiological levels of *Bcs1*.These are typical markers of *BCS1L-*linked CIII deficiency and, more generally, of mitochondrial disease. We showed that this very severe clinical phenotype is linked to a profound defect in complex III amount/activity and accumulation of intermediate CIII assemblies, closely resembling what was previously observed in patients. Interestingly, the parallel reduction in CI activity confirms observations from patients suffering from complex III deficiency. In fact, the instability of CI is a well-known phenomenon resulting from faulty CIII biogenesis in mammals [1, 33–38], as the assembly of the respiratory chain complexes appears to be strictly interconnected [39]. The severe effects described above are triggered by knocking-down *Bcs1* expression by 80%, without completely abolishing its function. This observation further supports the crucial role of Bcs1 in metazoans and possibly explains why no loss of function alleles of *BCS1L* have ever been described in homozygous state. We also established a fundamental role of Bcs1 in the brain and muscle (mesodermal derivatives), since the specific silencing of the gene in such tissues was sufficient to compromise their function, phenocopying features observed in patients, including encephalopathy and muscle hypotonia. B=Notably flies partially missing Bcs1 in CNS and mesodermal derivatives also died early. In addition, even a reduction in *Bcs1* expression restricted to a specific population of neurons (dopaminergic) caused age-related neuromotor dysfunction. Lastly, we knocked-down the expression of *Bcs1* in the fat body, i.e. the insects’ functional homolog of liver and adipose tissue of mammals. Any early-onset deleterious effect of *Bcs1* silencing was excluded, especially during development. Nevertheless, we observed signs of mitochondrial dysfunction in the tissue (impaired β-oxidation), leading to slow progression of phenotype over time, eventually resulting in early death. Thus, in contrast to humans and similar to mice, flies are not significantly affected by *Bcs1* dysfunction in the fat body, suggesting that physiological differences between species might explain why patients suffering from GRACILE syndrome exhibit severe liver disease. Indeed, in the GRACILE mouse liver, disease does not play a prominent role in the development of the clinical phenotype. This is in line with our observations, as dysfunction of *Drosophila Bcs1* in the fat body appears to have much less severe effects than in other tissues such as brain and muscle. Taken together, these observations point out to the possibility that liver-disease might be a specific effect of *BCS1L* dysfunction only in human GRACILE syndrome.

In conclusion, we have provided an extensive genetic and functional characterization of *Bcs1* in *Drosophila melanogaster*. In addition, we have validated and characterized in depth for the first time a non-mammalian animal model of *BCS1L*-related mitochondriopathy, phenocopying and mimicking several patients’ features. *Drosophila* is a promising tool to study the pathophysiology of *BCS1L*-related mitochondrial complex III deficiency, and to test novel treatment strategies, although the puzzling results of the GRACILE mutation in young mice plus the consistent absence of homozygous loss of function mutations of BCS1 in humans and the severity of partial loss of Bcs1 in flies should prompt to hypothesize an additional, yet unknown role of BCS1 that can account for its dispensability in CIII biogenesis in young mice, in contrast with its indispensability in humans and flies.

## Materials and methods

### Fly stocks and maintenance

Flies were raised on standard cornmeal medium and maintained at 23 °C, 70% relative humidity on a 12-h-light and 12-h-dark cycle. The UAS fly strain (y[1] v[1]; P{y[+ t7.7] v[+ t1.8] = TRiP.HMC03437}attP40, line ID 51863) used to perform *Bcs1* post-transcriptional silencing (from here onwards qualified as *UAS-shBcs1*) was obtained from Bloomington Stock Center. An additional UAS fly strain (line ID 110810) used to perform *Bcs1* post-transcriptional silencing (from here onwards qualified as *UAS-Bcs1-IR*) was obtained from the Vienna Drosophila Resouce Center (VDRC). The following *gal4* lines were obtained from the Bloomington Drosophila Stock Center (BDSC): *elav*^*C155*^*-gal4* (abbr. *elav-gal4*) strain ID 3954, *how24b-gal4* > *TM3* (abbr. *how24b-gal4*) strain ID 1767, *ppl-gal4* strain ID 58,768, *act5c-gal4* > *CyO.GFP* (abbr. *act5c-gal4*) strain (Bloomington strain ID 4414). Control flies were obtained by crossing each specific *gal4* driver line with the genetic background flies (*w*^*1118*^, obtained from Bloomington Drosophila Stock Center).

### Analysis of development and genetic frequencies

Developmental analysis was performed by counting the number of eggs, pupae and adult flies in 25 × 95 mm plastic vials under a Leica MZ6 Stereo Microscope. For developmental analysis, 15 virgin females of the driver line were crossed with 7 males of the responder line. After 24 h premating, flies were placed in new vials for egg laying and removed after further 24 h. A minimum number of 250 eggs from 3 different tubes were counted. Genetic frequencies were estimated by counting balanced and unbalanced flies in the progeny. Expected frequencies were evaluated by counting balanced and unbalanced flies in the progeny resulting from the cross between balanced gal4 driver lines and *w*^*1118*^ genetic background line.

### RNA isolation, reverse transcription and qRT-PCR

Total RNA was extracted from 10 adults (whole body), 30 heads, 15 larval body wall preparations or 15 adult abdominal fat body preparations for each genotype (1:1 males-females) using TRIzol reagent (Thermo Fisher Scientific) according to the manufacturer’s instructions. First-strand cDNA synthesis was performed with the GoScript reverse transcriptase kit (Promega). qRT-PCRs were performed in triplicate using a Bio-Rad CFX 96 Touch System (Bio-Rad) using GoTaq qPCR SYBR Green chemistry (Promega). The 2^−∆∆^Ct (RQ, relative quantification) method was used to calculate the relative expression ratio. *Rp49* was used as endogenous control, and the oligonucleotides employed were Rp49-Fw (5′-ATCGGTTACGGATCGAACAA-3′) and Rp49-Rv (5′-GACAATCTCCTTGCGCTTCT-3′). *Bcs1* oligonucleotides used were Bcs1-Fw (5′-CTGAATGTTGCGCCAGAG-3′) and Bcs1-Rv (5′-GACGAATGCTGCGTCGAT-3′). *Impl3* (*Ldh*) oligonucleotides used were Impl3-Fw (5′-CATCATCCCCAAGCTGGTAG-3′) and Impl3-Rv (5′-CCAGGCCACGTAGGTCAT-3′). *Pfk* oligonucleotides used were Pfk-Fw (5′-CGACCTCATTGCAGAGACGA-3′) and Pfk-Rv (5′-ACCACTGCTTCTTCGGGATG-3′). *Hex-A* oligonucleotides used were HexA-Fw (5′-CAAAATCAGCGACAGCGACC-3′) and HexA-Rv (5′-GCGGGCTGTGAGTTGTAAGA-3′). *PyK* oligonucleotides used were PyK-Fw (5′-TCTTGGTGACTGGCTGAAGG-3′) and PyK-Rv (5′-GCCGTTCTTCTTTCCGACCT-3′). *Hoe2* oligonucleotides used were Hoe2-Fw (5′-AGTGGACCACGCTGCTCT-3′) and Hoe2-Rv (5′-ATCTCTCCACGCATTCCATC-3′).

### Analysis of larval locomotor activity

Locomotor activity was assessed using 60-mm Petri dishes with graph paper (0.25 cm^2^ grids) attached to the bottom. Larvae were put in the dish and their activity was recorded with a digital camera for 1 min. The number of lines crossed and peristaltic movements per minute were measured for each larva. A total number of 20 individuals per genotype were analysed.

### Climbing test

The climbing test was performed using a modified version of the countercurrent apparatus originally designed by Seymour Benzer [40]. Twenty flies were placed into the first tube, tapped to the bottom and allowed to climb a 10-cm distance for 10 s. The flies that reached the 10 cm distance were shifted to a second tube, tapped again to the bottom and allowed to climb for further 10 s. The procedure was repeated five times. Then, the number of flies in each tube was counted. Climbing indexes were calculated as the weighted average of flies in the different tubes, divided by five times the number of flies in the test. A minimum number of 60 individuals per sex and genotype were analysed.

### Lifespan assay

Flies were reared at standard low density, collected after hatching and divided into males and females over a 24-h window. Adults of the same sex were kept at a density of 10 per vial (for a total number of 50 individuals) at 23 °C. Flies were counted and transferred to fresh medium three times per week, with no anaesthesia.

### Isolation of mitochondria

Mitochondria were prepared by differential centrifugation as described previously [41] with minor modifications. Larvae were homogenized with a Dounce glass potter and a loose-fitting glass pestle in 10 mL of isotonic isolation buffer (225 mM mannitol, 75 mM sucrose, 5 mM HEPES, 1 mM EGTA, pH 7.4) with 1% BSA. Samples were centrifuged at 1000 × g (Eppendorf 5810R) at 4 °C for 10 min. The supernatant was filtered through a 100-μm strainer (Corning) and centrifuged at 6,000 × g at 4 °C for 10 min. The mitochondrial pellet was washed in 10 mL of isolation buffer and centrifuged at 6000 × g for 10 min. The wash was repeated using 10 mL of isolation buffer without BSA and centrifuged at 7000 × g for 10 min. The mitochondrial pellet was re-suspended in minimal volume of isolation buffer without BSA. Protein concentration was measured by the Bradford assay (Bio-Rad protein assay).

### Analysis of MRC activity

Prior to enzymatic MRC complex activity assays, isolated mitochondria were subjected to 3 freeze–thaw cycles in 10 mM ice-cold Tris hypotonic buffer (pH 7.6) performing three freeze–thaw cycles with liquid nitrogen to disrupt the mitochondrial membranes. The activities of mitochondrial respiratory chain complexes and citrate synthase (CS) were measured by spectrophotometry, as described previously [42], following modified versions of the procedures for the reduction of decylubiquinone and cytochrome *c* [43].

### Cell cultures, transfection and subcellular localization analysis

The *Drosophila* S2R + cell line is derived from a primary culture of late stage (20–24 h old) *D. melanogaster* embryos, and it was obtained from the Drosophila Genomics Resource Center (DGRC ID 150). S2R + cells grow at 25 °C without CO_2_ in Schneider’s medium (Thermo Fisher Scientific) with 10% heat-inactivated foetal bovine serum (FBS) (Euroclone) as a loose, semi-adherent monolayer, showing a doubling time of about 48 h. *CG4908* cDNA was cloned in pAc5-STABLE2-neo vector [44], fused with HA tag using KpnI and HindIII cloning sites introduced at the 5′ and 3′ of *CG4908* by PCR amplification carried out using the following primers: *Bcs1-Fw-KpnI* (5′-AGACCCCGGATCGGGGTACCCAAAATGACTTTGCCCGATCTTGT-3′), *Bcs1-Rv-HindIII* (5′-CTCTGCCCTCAAGCTTTTAAGCGTAATCTGGAACATCGTATGGGTAATTAATCTTTTTATTATGATC-3′). S2R + cells were transfected in 12-well plate using Effectene Transfection Reagent (Qiagen) according to the manufacturer’s instructions. After 24 h, cells were prepared for immunostaining and confocal microscopy, as described previously [4]. The coverslips were mounted with Vectashield mounting medium (Vector Laboratories), and images were taken with a Zeiss LSM700 confocal microscope at 100 × magnification. Primary antibodies used for immunostaining of *Drosophila* cells were mouse monoclonal anti-HA (1:300, Sigma H9658) and mouse monoclonal anti-ATP5A (1:200, Abcam ab14748). Secondary antibodies used were goat anti-mouse Alexa Fluor 488 (1:100, Thermo Fisher Scientific A28175), donkey anti-mouse Alexa Fluor 647 (1:200, Thermo Fisher Scientific A-31571).

#### BNGE

Isolated mitochondria were resuspended in 1.5 M aminocaproic acid, 50 mM Bis–Tris/HCl pH 7.0. The samples were solubilized with 4 mg of *n*-dodecyl β-d-maltoside (Sigma) per mg of protein. After 5 min of incubation on ice, samples were centrifuged at 18,000 × g at 4 °C for 10 min. The supernatant was collected and resuspended with Sample Buffer (750 mM aminocaproic acid, 50 mM Bis–Tris/HCl pH 7.0, 0.5 mM EDTA and 5% Serva Blue G). Native samples were separated using NativePAGE 3–12% Bis–Tris gels (Thermo Fisher Scientific) according to the manufacturer’s protocol.

### Western blot and immunodetection

BN-PAGE gels were transferred to PVDF membranes in Dunn bicarbonate buffer (10 mM NaHCO_3_, 3 mM Na_2_CO_3_) applying a constant voltage of 25 V at 4 °C for 1 h using a XCell II™ Blot Module (Thermo Fisher Scientific). For the immunodetection of specific protein targets, blotted PVDF membranes were blocked in 5% skimmed milk in PBS-T (0.1% Tween-20) at room temperature for 1 h and then incubated overnight at 4 °C with primary antibodies diluted in 3% BSA in PBS-T. After incubating the primary antibody, the PVDF membranes were washed three times with PBS-T for 10 min and incubated with the secondary HRP-conjugated antibody for 1 h at room temperature. Chemiluminescent signals were recorded using an Alliance Mini HD9 (UVITEC). The antibodies used were mouse monoclonal anti-ATP5A (Abcam, Ab14748) and rabbit polyclonal against *D. melanogaster* UQCR-C2 (custom-made and kindly provided by Dr. Edward Owusu-Ansah, Columbia University, NY). Densitometric analysis was performed using the Fiji software version 2.0.0.

### Lactate quantification

Lactate content was measured using the Lactate-Glo Assay system (Promega). Briefly, 10 3rd instar larvae were homogenized in 150 μL of mammalian cell lysis solution on ice and centrifuged at 14,000 × g at 4 °C for 5 min to remove insoluble material. The supernatant was diluted 1:10 in HBSS (Thermo Fischer Scientific) and quantified according to the manufacturer’s protocol. Lactate content was normalized by protein content assessed by Bradford method.

### TAG quantification

For TAG quantification, freshly dissected 14 abdomens of 3-day-old adults (1:1 males-females) were analysed by coupled colorimetric assay (CCA) as previously described [45]. TAG content was normalized by protein content, as assessed by the Bradford method.

### Morphological analysis of larval structures

Morphological analysis of larval mouth hooks was performed on whole-mount preparations, as described by Van Der Meer [46]. Samples were analysed with a Leica DMR microscope using 40 × DIC optics, and images were taken by a Nikon Eclipse 80i using a 40 × differential interference contrast (DIC) objective.

### Statistical analysis

Statistical analysis was performed with GraphPad Prism Software, version 8.2.1. Statistical tests and significance are described in the figure captions.

## Supplementary Information

Below is the link to the electronic supplementary material.

Supplementary file1 (DOCX 983 KB)

Supplementary file2 (MP4 5786 KB)

## Data Availability

Data sharing not applicable to this article as no datasets were generated or analysed during the current study.
