# Supplementary material for: Modelling of BCS1L-related human mitochondrial disease in Drosophila melanogaster
Source: J Mol Med (Berl). 2021 Jul 17;99(10):1471–85. doi: 10.1007/s00109-021-02110-1 (PMC8455400; doi:10.1007/s00109-021-02110-1)

**Figure** **S1:** **(A)** Relative percentage of egg to adult viability of ubiquitous *Bcs1* KD cross (*act5c-gal4>CyO.GFP x UAS-Bcs1-IR*) and control cross (*act5c-gal4>CyO.GFP x w^1118^*), calculated at three developmental stages (eggs, pupae and adults), (n *>* 250, Chi-square test 9.343 df(2), **p < 0.01). **(B)** Mendelian frequencies of adults obtained by crossing heterozygous *act5c-gal4>CyO.GFP* flies with homozygous *UAS-Bcs1-IR* flies (n = 267, Chi-square test 123.7, df(1), ****p ≤ 0.0001). **(C)** *Bcs1* expression levels normalized by *Rp49* reference expression levels and measured by qPCR in whole larvae, expressed as relative quantity of template in the sample (RQ) in ubiquitous KD larvae (*act5c-gal4>UAS-Bcs1-IR*) compared to control larvae (*act5c-gal4>+*). Data are plotted as mean ± S.D. (n = 3, Student’s t test ***p ≤ 0.001). **(D)** *Hoe2* expression levels normalized by *Rp49* reference expression levels and measured by qPCR in whole larvae, expressed as relative quantity of template in the sample (RQ) in ubiquitous KD larvae (*act5c-gal4>UAS-Bcs1-IR*) compared to control larvae (*act5c-gal4>+*). Data are plotted as mean ± S.D. (n = 3, Student’s t test not significant). **(E)** Blue-native gel electrophoresis (BNGE) analysis of MRC complexes in DDM-solubilized isolated mitochondria from control (*act5c-gal4>+*) and *Bcs1* KD larvae (*act5c-gal4>UAS-Bcs1-IR*). **(F)** BNGE, Western blot and immunodetection of DDM-solubilized mitochondria from *Bcs1* KD (*act5c-gal4>UAS-Bcs1-IR*) and control (*act5c-gal4>+*) flies with antibodies against a CIII subunit (UQCR-C2) and a CV subunit (ATP5A) as loading control. Bands were quantified by measuring the integrated density of the signal. Data are plotted as mean ± S.D. (n = 3, Student’s t test *p ≤ 0.05).


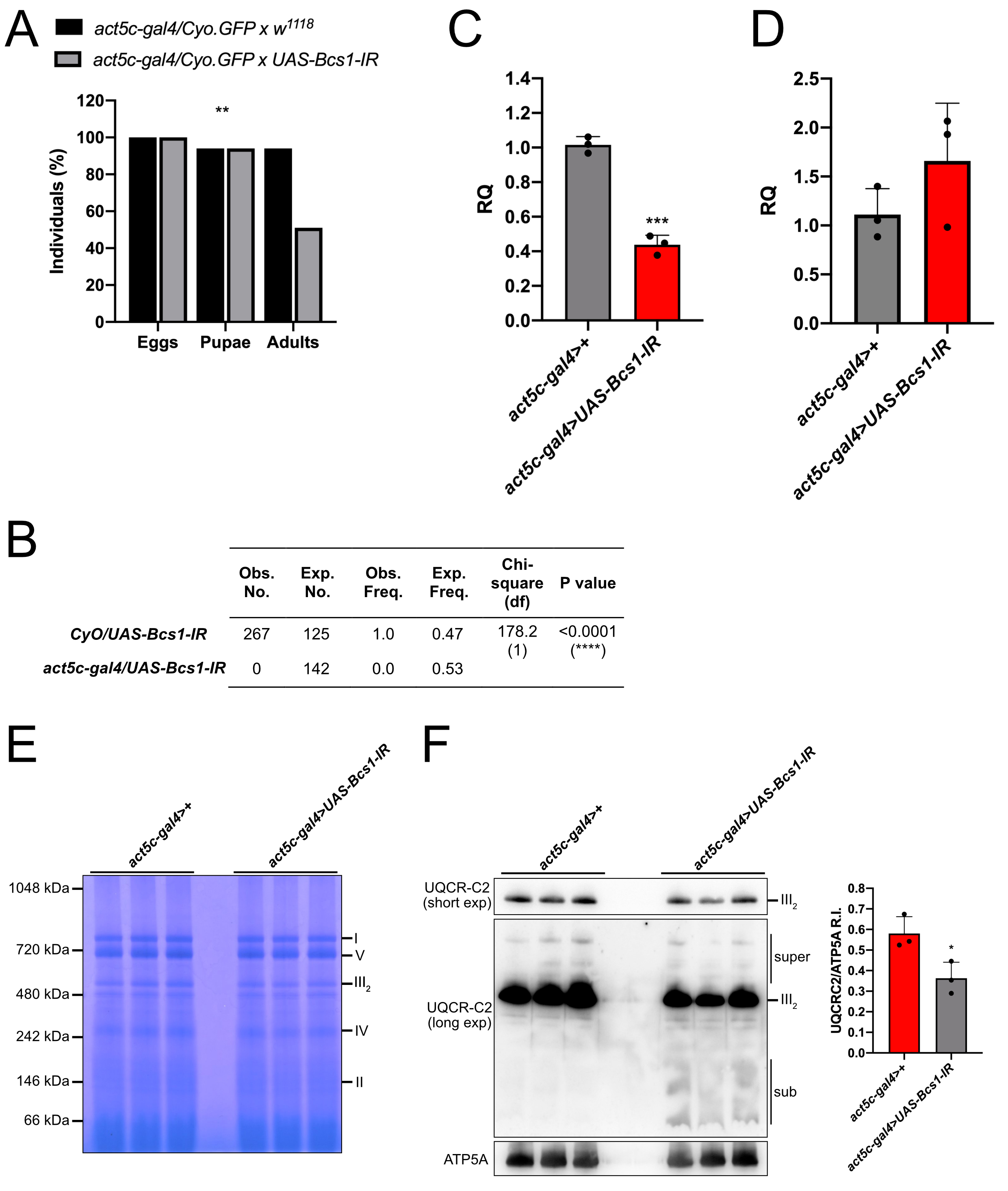

Supplement: Supplementary file 1 — Supplementary file1 (DOCX 983 KB) [file 109_2021_2110_MOESM1_ESM.docx]
